# Osteoprotegerin Inhibits Aortic Valve Calcification and Preserves Valve Function in Hypercholesterolemic Mice

**DOI:** 10.1371/journal.pone.0065201

**Published:** 2013-06-06

**Authors:** Robert M. Weiss, Donald D. Lund, Yi Chu, Robert M. Brooks, Kathy A. Zimmerman, Ramzi El Accaoui, Melissa K. Davis, Georges P. Hajj, M. Bridget Zimmerman, Donald D. Heistad

**Affiliations:** 1 Division of Cardiovascular Medicine, University of Iowa Carver College of Medicine, Iowa City, Iowa, United States of America; 2 Department of Biostatistics, College of Public Health, University of Iowa, Iowa City, Iowa, United States of America; 3 Department of Pharmacology, University of Iowa Carver College of Medicine, Iowa City, Iowa, United States of America; Scuola Superiore Sant'Anna, Italy

## Abstract

**Background:**

There are no rigorously confirmed effective medical therapies for calcific aortic stenosis. Hypercholesterolemic *Ldlr* ^−/−^ *Apob* ^100/100^ mice develop calcific aortic stenosis and valvular cardiomyopathy in old age. Osteoprotegerin (OPG) modulates calcification in bone and blood vessels, but its effect on valve calcification and valve function is not known.

**Objectives:**

To determine the impact of pharmacologic treatment with OPG upon aortic valve calcification and valve function in aortic stenosis-prone hypercholesterolemic *Ldlr* ^−/−^ *Apob* ^100/100^ mice.

**Methods:**

Young *Ldlr* ^−/−^ *Apob* ^100/100^ mice (age 2 months) were fed a Western diet and received exogenous OPG or vehicle (N = 12 each) 3 times per week, until age 8 months. After echocardiographic evaluation of valve function, the aortic valve was evaluated histologically. Older *Ldlr* ^−/−^ *Apob* ^100/100^ mice were fed a Western diet beginning at age 2 months. OPG or vehicle (N = 12 each) was administered from 6 to 12 months of age, followed by echocardiographic evaluation of valve function, followed by histologic evaluation.

**Results:**

In Young *Ldlr* ^−/−^ *Apob* ^100/100^ mice, OPG significantly attenuated osteogenic transformation in the aortic valve, but did not affect lipid accumulation. In Older *Ldlr* ^−/−^ *Apob* ^100/100^ mice, OPG attenuated accumulation of the osteoblast-specific matrix protein osteocalcin by ∼80%, and attenuated aortic valve calcification by ∼ 70%. OPG also attenuated impairment of aortic valve function.

**Conclusions:**

OPG attenuates pro-calcific processes in the aortic valve, and protects against impairment of aortic valve function in hypercholesterolemic aortic stenosis-prone *Ldlr* ^−/−^ *Apob* ^100/100^ mice.

## Introduction

Aortic valve stenosis afflicts <1% of the U.S. population, but for age ≥75 the prevalence is 2.8%. [1] Although several medical therapies showed early promise for slowing the progression of aortic valve stenosis, [2], [3] no therapy to date has demonstrated efficacy in prospective randomized clinical trials. [4], [5] Thus, replacement of the aortic valve is the only accepted treatment for patients with symptomatic aortic stenosis. [6].

In adults, acquired aortic stenosis is almost always “calcific”, characterized by formation and coalescence of calcium-phosphate salts in the valve and its supporting structures. [7] Aortic stenosis arises from a series of orchestrated processes including increased oxidant stress, inflammation, transdifferentiation of valve interstitial cells, and processes which resemble mineralization of the skeleton. [8], [9].

Receptor activator of nuclear factor κB (RANK) is a member of the tumor necrosis factor receptor-like family of cytokines. Binding of RANK to its ligand (RANKL) promotes skeletal demineralization, [10] but increases calcification in blood vessels. [11] Osteoprotegerin (OPG) inhibits RANK:RANKL signaling by acting as a “decoy receptor” for RANKL, rendering it inaccessible for RANK binding and downstream signaling. [10] Mice deficient in OPG develop severe osteoporosis and prominent vascular calcification at an early age. [12] In hypercholesterolemic *Ldlr* ^−/−^ mice, exogenous OPG does not inhibit development of atherosclerosis, but prevents calcification of the aorta, [13] by mechanisms which have not been fully elucidated.

*Ldlr* ^−/−^mice which express apolipoprotein B100 only (*Ldlr* ^−/−^ *Apob* ^100/100^, or “LA mice”) are prone to develop calcific aortic valve stenosis. [14] Reversal of hypercholesterolemia during early adulthood prevents progression of aortic valve disease in *Ldlr* ^−/−^ *Apob* ^100/100^/*Mttp* ^fl/fl^ *Mx1Cre* ^+/+^ (Reversa) mice. [15] However, the independent causal contribution of calcification *per se* toward functional impairment in cardiovascular tissue has not been experimentally confirmed. Indeed, in old Reversa mice, reversal of hypercholesterolemia produces marked reduction in aortic valve calcium content, but does not improve aortic valve function. [16] Thus, reduction of aortic valve calcium alone does not necessarily translate to improved valve function.

The goals of the present study were to determine the effect of treatment with exogenous OPG upon calcification in the aortic valve, and to test the hypothesis that attenuation of aortic valve calcification preserves aortic valve function in hypercholesterolemic mice. Here we report that OPG attenuates aortic valve calcification by inhibition of the activity of osteoblast-like cells, and consequently attenuates aortic valve dysfunction in stenosis-prone LA mice.

## Methods

### Ethics Statement

All studies were approved the Institutional Animal Care and Use Committee at the University of Iowa (PHS Animal Welfare Assurance #A3021-01). All procedures were designed and performed in a manner so as to minimize pain and discomfort.

A detailed description of the experimental methods can be viewed in Text S1.

### Experimental Strategy

**Figure 1:**
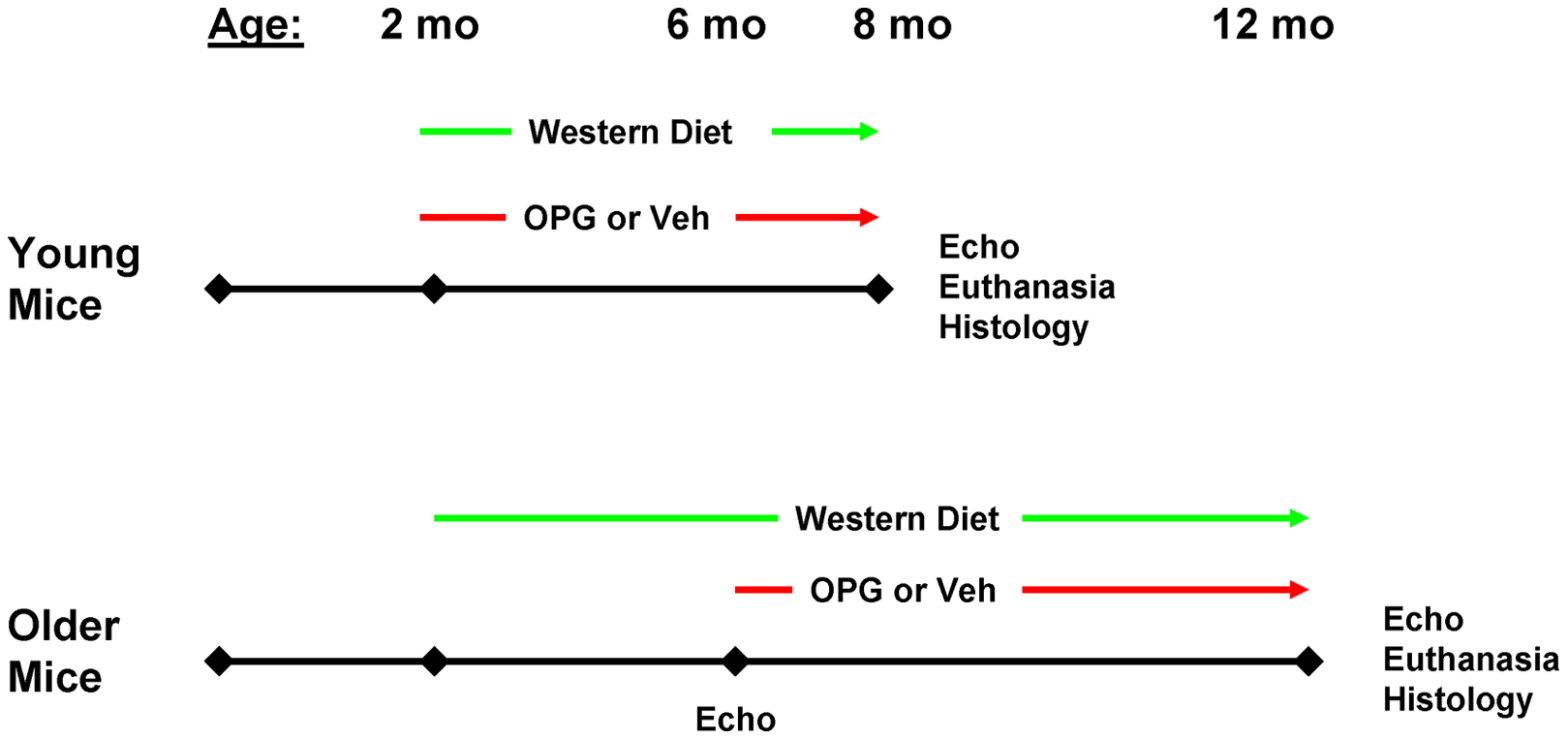
Experimental Strategy.

A schematic of the experimental strategy is shown in Figure 1. Mice were studied at two ages. “Young” LA mice, were fed a Western diet and received injections of OPG three times per week, beginning at 2 months of age, and are designated “Young OPG-LA” (N = 12). A group of littermate Young LA mice were also fed a Western diet, but received injections of phosphate buffer vehicle only, and are designated “Young Veh-LA” (N = 12). At 8 months of age, Young mice underwent echocardiography, followed by euthanasia, collection of blood, and histologic studies.

“Older” LA mice were fed a Western diet beginning at 2 months of age. At 6 months of age, the mice underwent echocardiography and then received injections of OPG (“Older OPG-LA”, N = 12) or vehicle (“Older Veh-LA”, N = 12) 3 times per week. At 12 months of age, Older mice underwent echocardiography, followed by euthanasia, collection of blood, and histologic studies.

### Histologic Studies

Valve tissue samples from Young mice were quantitatively analyzed for evidence of “early” disease: lipid accumulation, osteogenic transformation of valve cells, and valve calcification. Valve tissue samples from Older mice were quantitatively analyzed for evidence of fibrocalcific aortic valve disease, i.e. collagen accumulation and calcification, and also for lipid content. Immunohistochemical studies were performed to quantitate levels of osteocalcin, a bone matrix-specific protein, and for monocyte chemo-attractant protein-1, a signal for valve inflammation. Dihydroethidine fluorescence was utilized to quantitate superoxide levels in valve tissue.

### Gene Expression

Methods for determination of the effects of exogenous OPG upon endogenous expression of OPG, RANK, RANKL, and TRAIL are described in Text S1.

### Echocardiography

Aortic valve function was quantified using an echocardiographic method that we have validated by hemodynamic measurements, as described previously. [14].

### Statistical Analysis

All group data are reported as mean ± SE. The primary analysis consisted of comparison of findings between LA mice treated with OPG *vs.* LA mice treated with vehicle. Echocardiographic and quantitative histologic parameters were first subjected to a Shapiro-Wilk test, to determine whether individual data points conformed to a Gaussian distribution sufficiently to undergo unpaired t-testing. Results of Shapiro-Wilk testing are shown in Table S1. Statistical significance of the effect of treatment with OPG vs. treatment with Veh, for parameters conforming to a Gaussian distribution was assessed and reported using unpaired t-testing. Statistical significance of histologic findings which did not meet Shapiro-Wilk criteria was assessed using Wilcoxon rank sum testing. Statistical significance for the effect of age upon aortic valve function was assessed within each treatment group using paired t-testing. In each case, statistical significance was present when p<0.05.

## Results

### Morphometric and Metabolic Parameters

(Table 1) Exogenous OPG had no effect on body mass, plasma cholesterol, or phosphorus in Young or Older LA mice. Plasma calcium concentration was lower in Older mice than in Young mice, but was not affected by OPG treatment. OPG reduced serum *Trap*5, which indicates systemic antagonism of RANKL signaling in Young mice and Older mice. Exogenous OPG had no effect on expression of endogenous OPG, RANKL, RANK, or tumor necrosis factor-related apoptosis-inducing ligand (TRAIL) in the aorta of LA mice (Figure S1).

**Table 1:** Morphometric and metabolic parameters.

	Young Mice		Older Mice	
	Veh-LA	OPG-LA	Veh-LA	OPG-LA
Body Mass (g)	36±2	36±2	37±3	35±3
% Male	46	43	42	50
Plasma Cholesterol (mg/dl)	777±82	826±102	807±115	787±175
Plasma Calcium (mg/dl)	10.2±0.3	10.7±0.6	7.3±0.3†	7.5±0.3†
Plasma Phosphate (mg/dl)	9.8±0.5	10.1±0.8	9.8±0.7	8.3±1.3
Plasma TRAP-5b (U/l)	20±2	6.5±0.4*	3.1±0.2†	1.6±0.1* †

**Veh-LA:** vehicle-treated Ldlr^−/−^Apob^100/100^ mice; **OPG-LA**: osteoprotegerin-treated Ldlr^−/−^Apob^100/100^ mice.

* p<0.05 vs. Veh-LA;

† p<0.05 vs. Young mice.

### Valve Lipid Content

**Figure 2:**
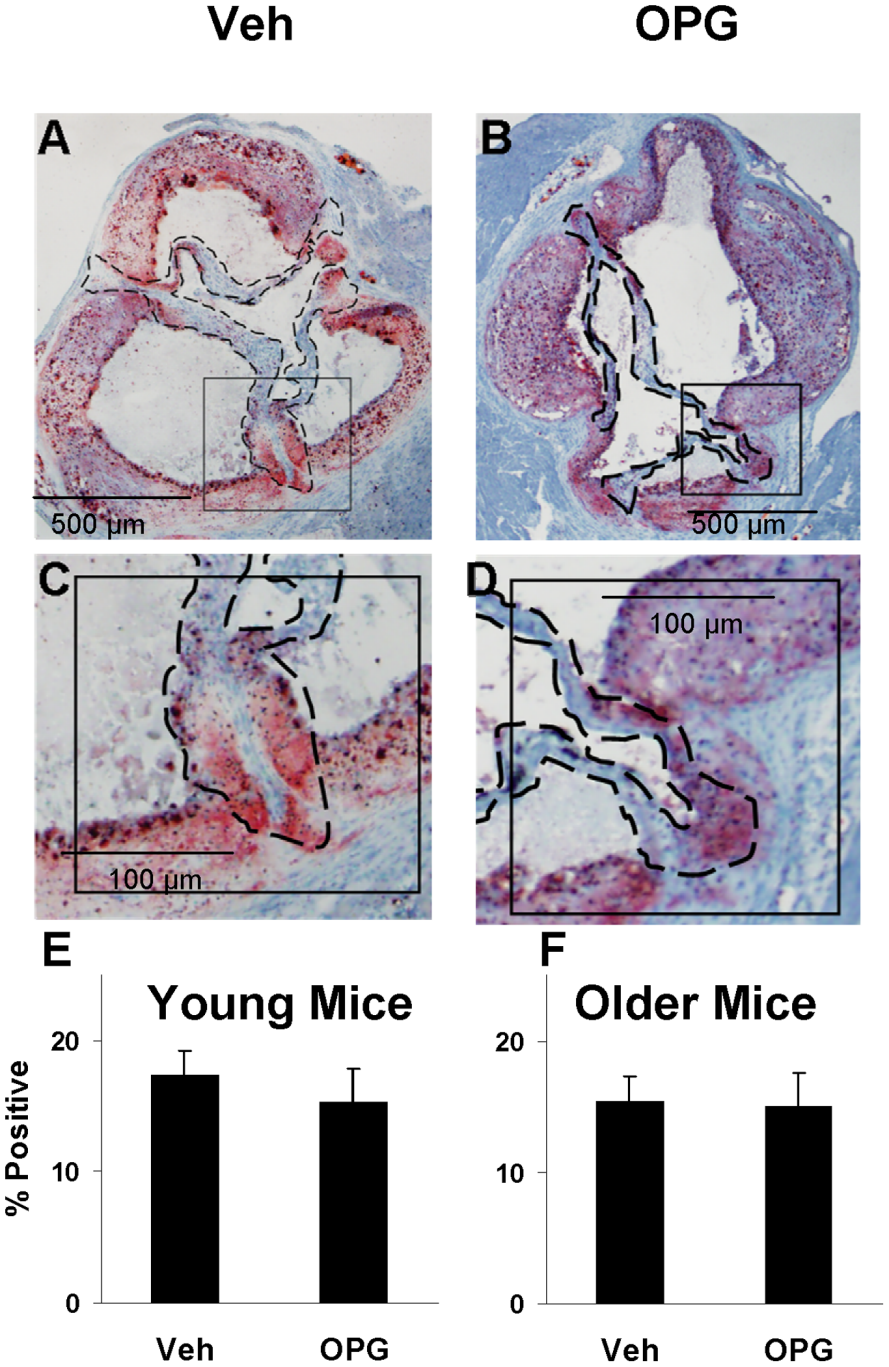
Lipid in the aortic valve. Oil Red-O staining is equally abundant in Older vehicle-treated mice (A,C) and OPG-treated Older mice (B,D). Group data for Younger mice (E) and Older mice (F). N = 7–8; p = NS for Veh vs. OPG.

Valve lipid content was not significantly affected by OPG in Young mice or Older mice (Figure 2).

### Osteogenic Transformation in the Aortic Valve

**Figure 3:**
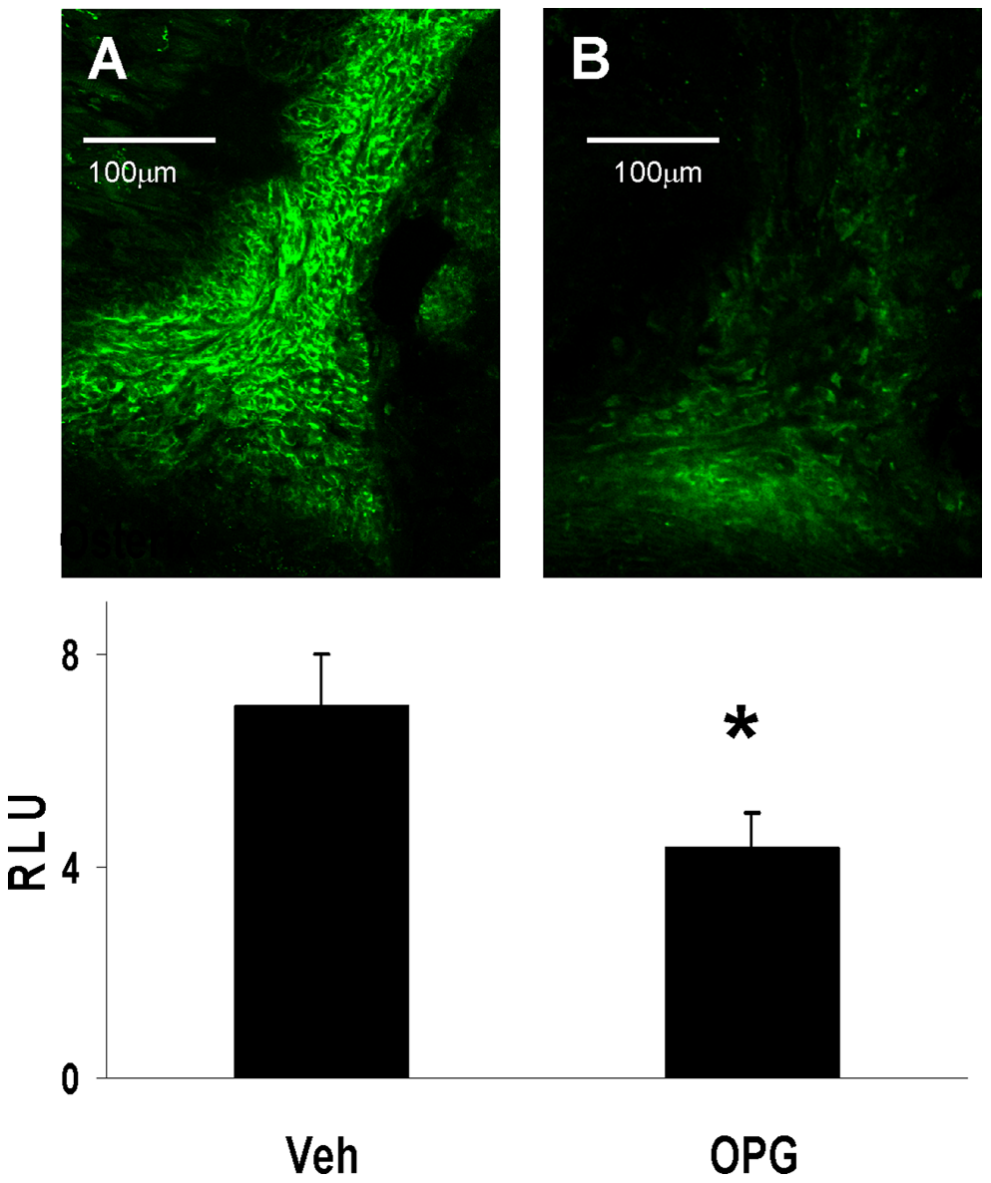
Immunofluorescent staining for osterix in the aortic valve in Young LA mice. There is abundant staining (green) at the cusp base in a vehicle-treated mouse (**A**), but less staining in the cusp base from an OPG-treated mouse (**B**). N = 12. *p<0.05 for Veh vs. OPG; **RLU** relative light units.

Osterix, a marker for transdifferentiation to bone synthesis-competent osteoblast-like cells, [17], [18] was identified in Young LA mice. OPG decreased osterix expression by about 40% (Figure 3).

### Bone-like Components in the Aortic Valve

**Figure 4:**
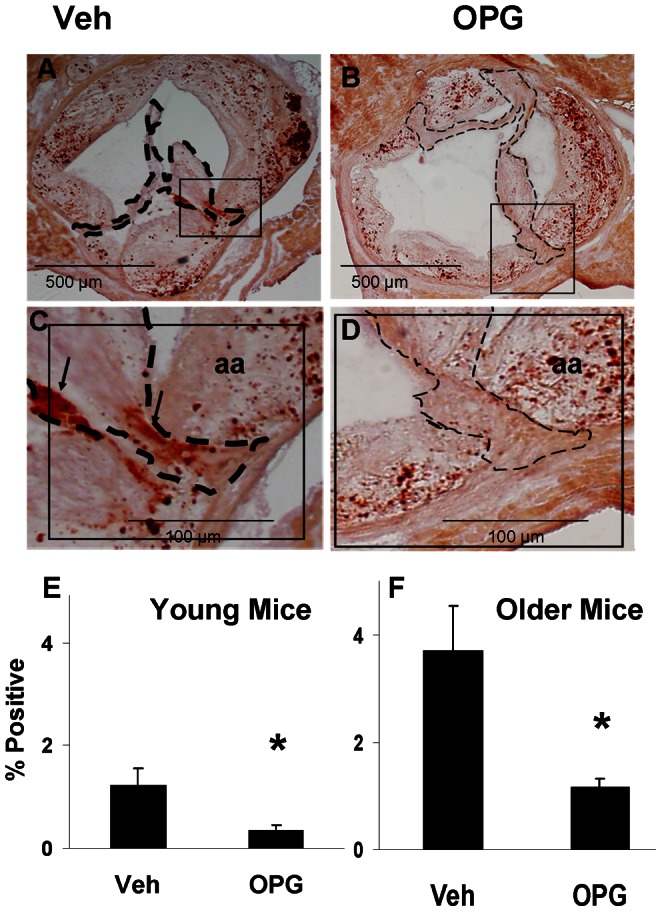
Calcification in the aortic valve. Alizarin Red staining in a valve from an Older vehicle-treated mouse (**A,C**) demonstrates bright red staining, indicating valve calcification (arrows). Valve cusps are thickened in an Older OPG-treated mouse, but are minimally calcified (**B,D**). Dashed borders contain valve cusps, with care taken to exclude the aortic annulus (aa). Group data for valve calcification in Young mice (E) and Older mice (F). *p<0.05 Veh vs. OPG, N = 12.

**Figure 5:**
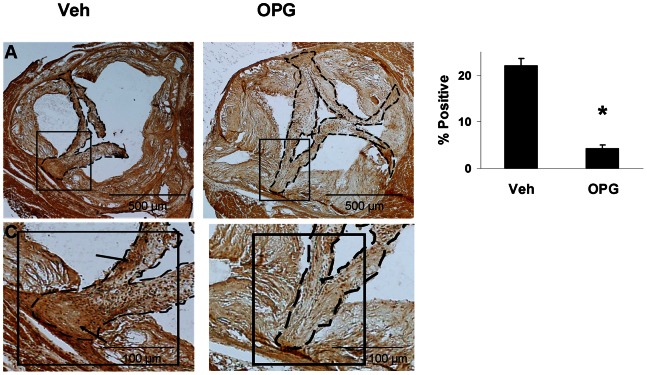
Immunostaining for osteocalcin in the aortic valve in Older mice. In vehicle-treated mice (**A,C**), osteocalcin (dark brown) is abundant near the cusp base (arrows). There is only scant staining in the valve of an OPG-treated mouse (**B,D**). N = 4. *p<0.05 Veh vs. OPG.

OPG attenuated calcification of valve tissue in Young mice and in Older mice by about 70% (Figure 4). OPG also attenuated accumulation of the bone matrix-specific protein, osteocalcin, in the aortic valve of Older mice by about 80% (Figure 5).

### Pro-inflammatory Signaling in Older Mice

**Figure 6:**
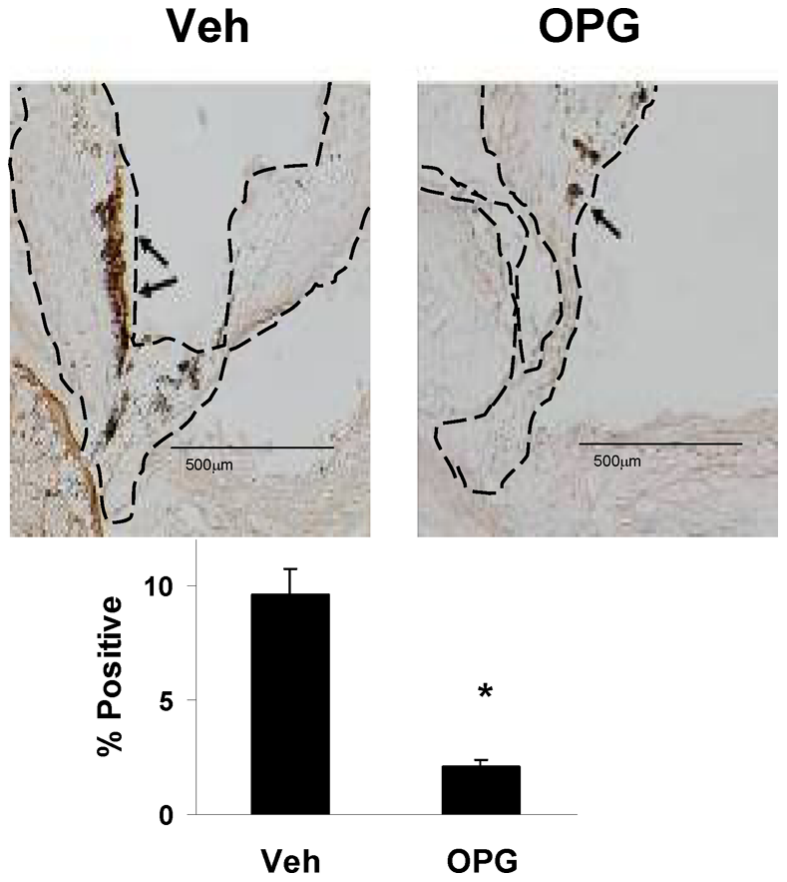
Immunostaining for monocyte chemo-attractant protein-1 (MCP-1) in Older LA mice. OPG decreased levels of MCP-1 in the aortic valve. N = 11 (Veh) and N = 6 (OPG). *p<0.05 for Veh vs. OPG.

Expression of the pro-inflammatory chemokine, monocyte chemo-attractant protein-1, was observed in valves from Older LA mice, and was inhibited by about 75% by OPG treatment (Figure 6).

### Oxidant Stress

Superoxide was examined in the aortic valve in Older mice. Superoxide levels were not affected by OPG treatment (Figure S2).

### Valve Fibrosis

**Figure 7:**
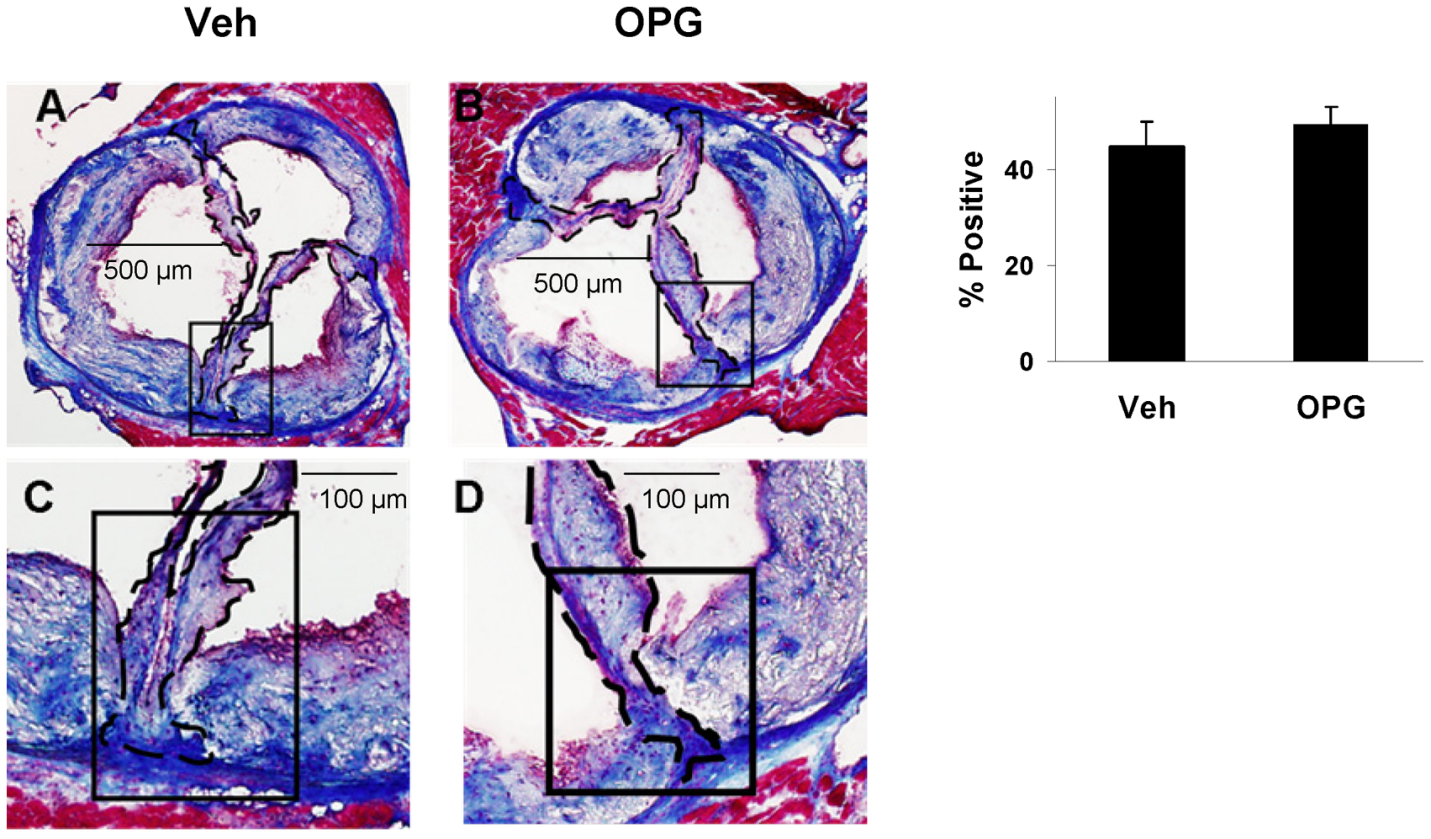
Masson’s Trichrome staining for collagen in Older mice. Collagen (dark blue) was equally abundant in valves of vehicle-treated mice (**A,C**) and OPG-treated mice (**B,D**). N = 12; p = NS for Veh vs. OPG.

OPG did not attenuate fibrosis of the valve, assessed using Masson’s Trichrome staining, significantly in Older LA mice (Figure 7). Because of the importance of differentiating the selective effect of OPG on valve calcification, but not on fibrosis, we performed confirmatory studies using Picrosirius Red staining for valve fibrosis, and again found no significant effect of OPG treatment upon valve fibrosis (Figure S3).

### Aortic Valve Function

There was a trend toward improved valve function in Young OPG-LA mice, compared to Young Veh-LA mice, but the finding did not achieve statistical significance (cusp separation = 1.0±0.05 mm for OPG-LA vs. 0.89±0.05 mm for Veh-LA; p = 0.13).

**Figure 8:**
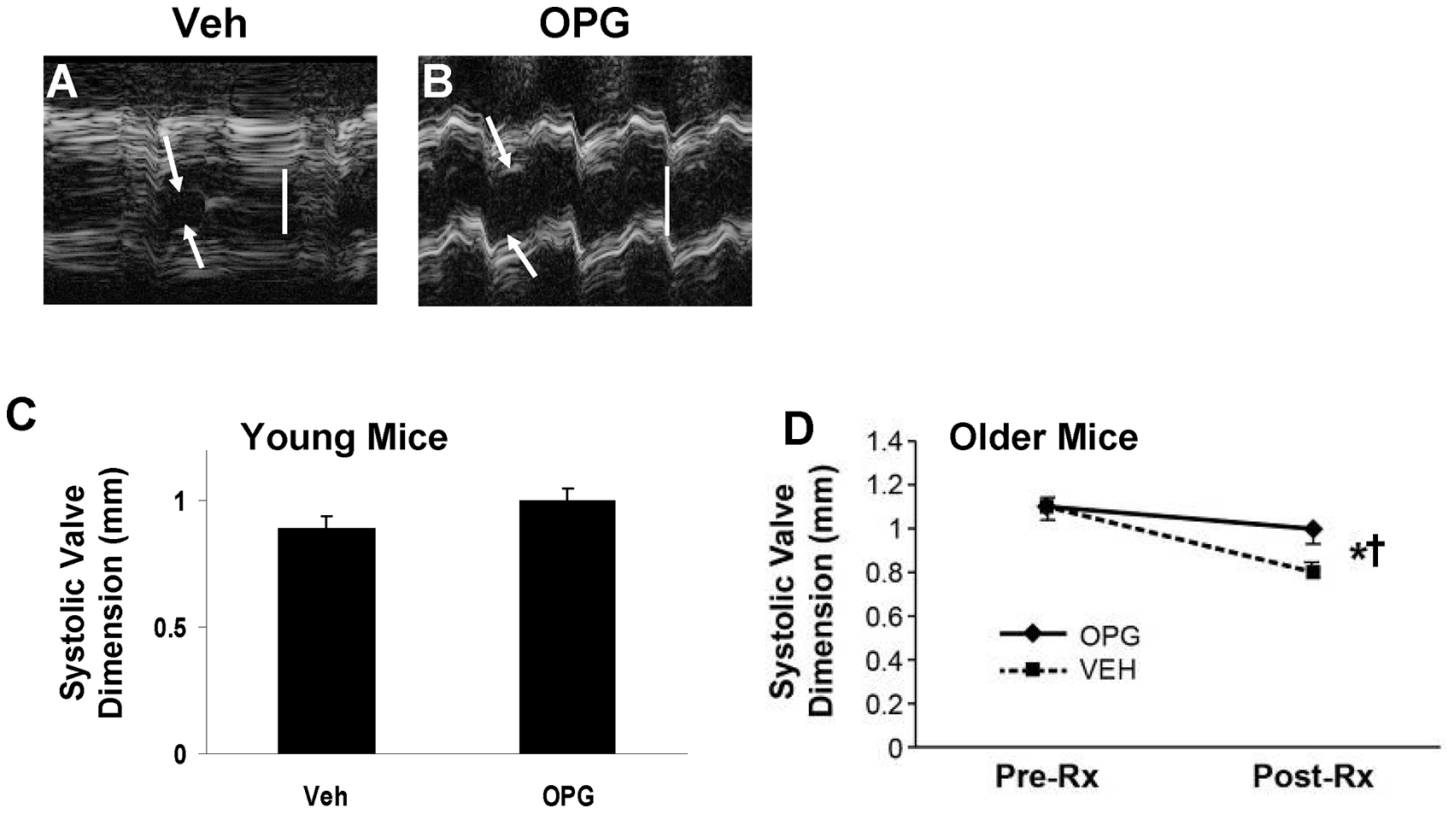
Aortic valve function. **A,B**: M-mode echocardiograms from Older mice depict aortic valve systolic dimension (arrows). Vertical white bar = 1 mm. **C**: Aortic systolic valve dimension in Young Mice studied at age 8 mo. N = 12, p = NS. **D**: Aortic valve systolic valve dimension in Older LA mice before (Pre-Rx, age 6 mo.) and after (Post-Rx, age 12 mo.) treatment with VEH or OPG, N = 12. *p<0.05 for VEH *vs*. OPG. † p<0.05 for 12 mo. vs. 6 mo. for comparisons within each treatment group.

In Older mice, systolic aortic cusp separation was similar in Veh-LA mice and OPG-LA mice, prior to initiation of treatment (Figure 8). Following 6 months of treatment with OPG or vehicle, aortic cusp separation decreased in both Veh-LA mice and OPG-LA mice. However, the decrease in aortic valve function was significantly attenuated in OPG-LA mice, compared to Veh-LA mice. Thus, by 12 months of age, aortic valve function was significantly better in OPG-LA mice, compared to Veh-LA mice (Figure 8). There was no significant difference in impairment of valve function between males and females in either treatment group, nor in the entire cohort of Older LA mice.

## Discussion

The most important finding of this study is that exogenous OPG protects aortic valve function in hypercholesterolemic *Ldlr* ^−/−^ *Apob* ^100/100^ mice, which are prone to develop calcific aortic stenosis. To our knowledge, this is the first report of a pharmacologic intervention that attenuates impairment of aortic valve function in stenosis-prone subjects of any species, in a randomized prospective study.

Protection of aortic valve function by OPG is accompanied by profound attenuation of valve calcification, but not by attenuation of valve fibrosis or lipid deposition. A major mechanism by which OPG attenuates valve calcification is by inhibition of bone-like synthesis in the valve. Synthesis-competent osteoblasts secrete calcium salts and matrix proteins into the extracellular matrix, resulting in bone-like deposits in diseased aortic valves. OPG strongly suppresses levels of osteocalcin, a protein that is specific to bone-like tissue, in approximate proportion to suppression of valve calcification.

A major mechanism by which OPG attenuates bone-like synthesis in the aortic valve is by inhibition of osteogenic transformation. Transformation of valve cells to bone-like, synthesis-competent (osterix-expressing [17]), cells was strongly inhibited by OPG during the “early” phase of valve disease, in Young mice.

RANKL, which is expressed minimally in normal aortic valves, is upregulated in the presence of calcific aortic stenosis. [19] In cultured aortic valve myofibroblasts, exogenous RANKL accelerates progression of an osteogenic phenotype. [19] In cultured vascular smooth muscle cells, exogenous RANKL promotes extracellular mineralization via a bone morphogenetic protein-4 dependent mechanism. [20] OPG is a decoy receptor with very high affinity for RANKL. [10] Thus, mechanisms by which OPG results in reduced valve calcification can be deduced from its inhibition of RANK:RANKL signaling.

Valve calcification, however, is a complex phenotype which accumulates as the result of multiple processes, including local hemodynamic stresses and systemic mechanisms. Thus, it is not likely that the effects of OPG, or the disease itself, are limited to a single linear signaling sequence in a single cell type. Antagonism of RANKL by OPG may act to decrease monocyte infiltration into hypercholesterolemic valve tissue. We found that OPG treatment significantly reduced expression of monocyte chemo-attractant protein-1 in valve tissue. Because tissue monocytes differentiate into macrophages, our finding is consistent with an anti-inflammatory component of OPG treatment. Thus, the association between attenuation of valve calcification and protection of aortic valve function by OPG may not necessarily indicate mechanistic linkage between the two findings.

In adult humans, acquired aortic stenosis is typically calcific. Evidence for a causal role of calcification in the process of progressive aortic valve dysfunction has heretofore been based principally on the very strong association between heavy calcification and aortic stenosis. However, inflammation, lipid deposition, and fibrosis are also present in stenotic aortic valves. [7] The results of the present study indicate that an intervention directed at attenuation of valve calcification, initiated prior to development of overt disease, inhibits the development of valve dysfunction, and thus suggests a role for calcification in the processes that lead to aortic valve stenosis.

### Summary

Taken together, our findings indicate that OPG strongly attenuates osteogenic transformation of valve cells, bone-like matrix synthesis, and calcification in aortic valves of stenosis-prone hypercholesterolemic mice. Aortic valve function is protected by a pharmacologic treatment, OPG, which is a unique finding.

### Study Limitations

LA mice are prone to develop aortic stenosis due to longstanding hypercholesterolemia, which is a risk factor for aortic stenosis in humans. It is not known whether OPG treatment would have a similar therapeutic effect in aortic valve disease associated with other clinical risk factors.

We provide extensive data supporting the anti-calcific action of OPG, which implicates antagonism of RANKL signaling as its mechanism of action. RANKL is produced by macrophages, and also by T-lymphocytes, which are known to be active in diseased aortic valves. [21] Because RANKL signaling putatively can occur via both cell-bound and soluble forms, we are not able to exclude an effect of OPG upon T-cell derived RANKL as a factor contributing to its anti-calcific effect.

Because of the importance of oxidant stress in cardiovascular disease processes arising from hypercholesterolemia, we measured superoxide levels in the aortic valves of LA mice. The assay requires the use of frozen sections, which were also utilized for histologic and Immunohistochemical studies. We also quantitated valve lipid content, which is not reliable after formalin fixation. Thus, our experimental strategy, which utilized frozen sections, resulted in loss of some tissue due to crush artifact, and probably also resulted in some loss of fine detail in the histologic sections, compared to results that may have been obtained using formalin fixation of valve tissue.

Because the aortic valve in mice is tiny, limited tissue was available for histologic analysis from each mouse. Thus, not all histologic parameters could be determined for mice from each age group. For that reason, tissue from Young mice was utilized to study “early” mechanisms of aortic valve disease: lipid accumulation, osteogenic transformation of valve cells, and early valve calcification. Tissue from Older mice was used to study the impact of OPG treatment upon factors putatively involved in valve dysfunction: valve calcification, fibrosis, lipid accumulation, and also upon mechanisms by which aortic valve disease may progress: inflammation and oxidant stress.

We treated mice using a single dose of OPG that was higher than has been utilized in other studies.^13^ Because of the duration and complexity of the experimental design, we are not able to report an optimal or minimum effective dose or duration for OPG. We note that administration of OPG did not affect basic morphometric or metabolic parameters in young mice or older mice (Table 1).

### Clinical Implications

The search for effective therapies to preserve valve function in patients with overt or incipient aortic stenosis has thus far been unsuccessful. [22] Calcific aortic stenosis, like osteoporosis, is a disease associated with aging and inflammation. [23] A therapeutic strategy designed to antagonize the effect of increased RANKL signaling upon tissue inflammation and mineralization, using the neutralizing monoclonal antibody denosumab, is safe and effective for patients with osteoporosis. [24] Thus, an approach with a similar mechanistic basis targeted at pathological dysregulation of mineralization in aortic valve tissue may offer opportunities for exploration of therapies for patients who are prone to develop aortic stenosis.

## Supporting Information

Figure S1 **Relative mRNA levels (normalized to β-actin) in aortas from Older mice were quantified by qRT-PCR. n = 11 for Veh, n = 6 for OPG.**
(TIFF)

Figure S2 **Superoxide in the aortic valve of Older mice. Values are shown as relative light units (RLU) of dihydroethidium fluorescence, a reporter for superoxide levels in tissue. p = NS.**
(TIFF)

Figure S3 **Picrosirius Red staining for fibrosis in the aortic valve from Older vehicle-treated LA mice (Veh, N = 6) and from Older OPG-treated LA mice (OPG, N = 4).** Fibrosis was quantitated in valve cusps (*),but not in valve annulus atheroma (A). p = NS.
(TIF)

Table S1 Statistical analysis of echocardiographic and histologic data.
(DOC)

Text S1 Detailed description of methods.
(DOC)
