# Supplementary figures and images for: Osteoprotegerin Inhibits Aortic Valve Calcification and Preserves Valve Function in Hypercholesterolemic Mice
Source: PLoS One. 2013 Jun 6;8(6):e65201. doi: 10.1371/journal.pone.0065201 (PMC3675204; doi:10.1371/journal.pone.0065201)

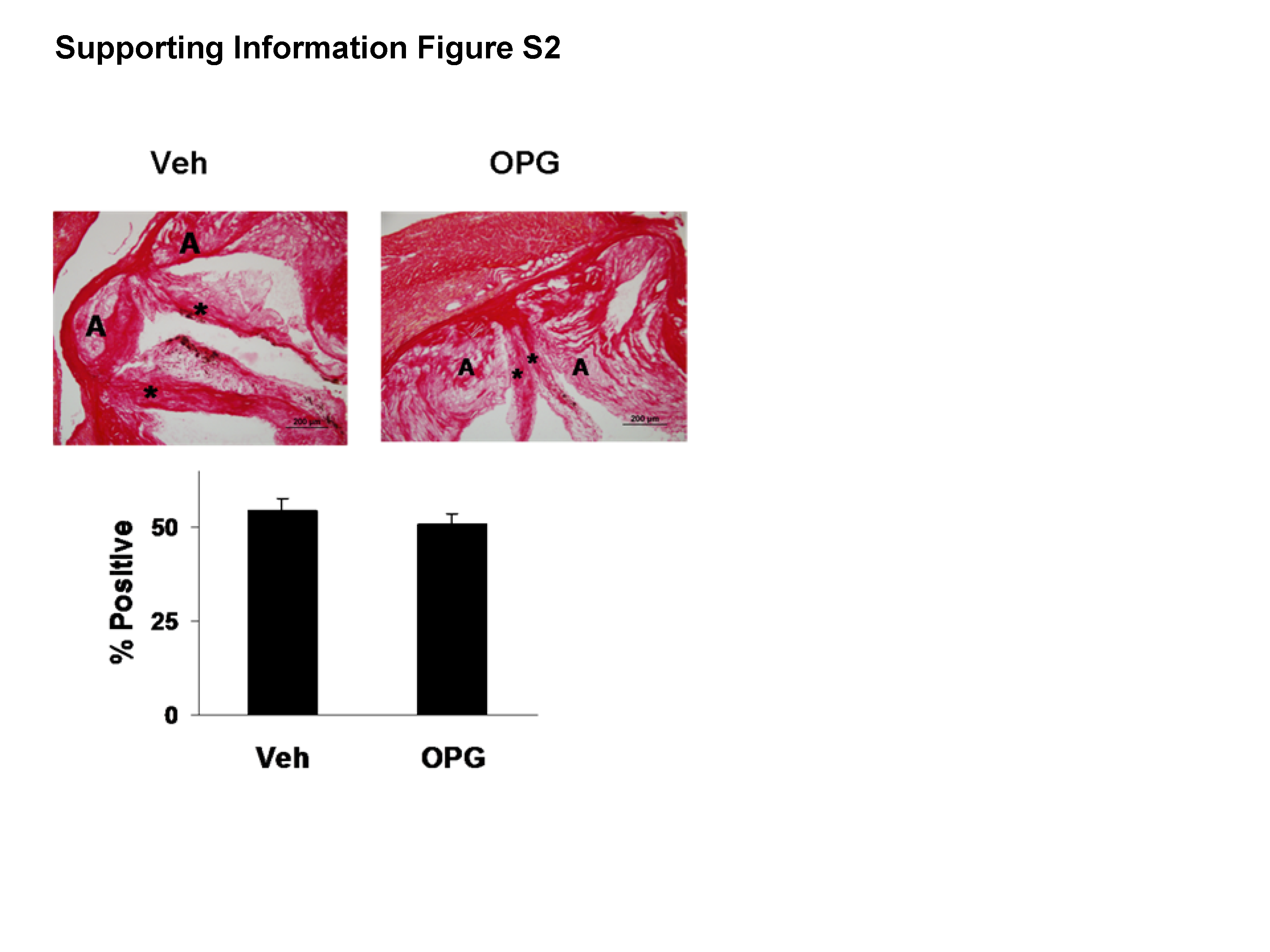

Supplement: Figure S3 — Picrosirius Red staining for fibrosis in the aortic valve from Older vehicle-treated LA mice (Veh, N = 6) and from Older OPG-treated LA mice (OPG, N = 4). Fibrosis was quantitated in valve cusps (*),but not in valve annulus atheroma (A). p = NS. (TIF) [file pone.0065201.s003.tif]
