# Supplementary material for: Osteoprotegerin Inhibits Aortic Valve Calcification and Preserves Valve Function in Hypercholesterolemic Mice
Source: PLoS One. 2013 Jun 6;8(6):e65201. doi: 10.1371/journal.pone.0065201 (PMC3675204; doi:10.1371/journal.pone.0065201)
Supplement: Table S1 — Statistical analysis of echocardiographic and histologic data. (DOC) [file pone.0065201.s004.doc]

**SUPPORTING INFORMATION Table S1**. Statistical Analysis of Echocardiographic and Histologic Data.

| **Variable** | **Gaussian Distribution?*** | **Wilcoxon rank sum test** | **T-test** |
| --- | --- | --- | --- |
| ACS | Yes |  | p=0.039 |
| AR | No | p=0.004 |  |
| MCP-1 | No | p=0.001 |  |
| MT | No | p=0.163 |  |
| ORO | Yes |  | p=0.886 |
| Ocn | Yes |  | p=0.039 |
| Osx | Yes |  | p=0.005 |

* Using Shapiro-Wilk testing. **ACS** aortic cusp separation; **AR** Alizarin Red staining for calcification; **MCP-1** monocyte chemoattractant protein-1; **MT** Masson’s Trichrome staining for collagen; **ORO** oil red-O staining for lipid; **Ocn** osteocalcin; **Osx** osterix
