# Supplementary material for: Osteoprotegerin Inhibits Aortic Valve Calcification and Preserves Valve Function in Hypercholesterolemic Mice
Source: PLoS One. 2013 Jun 6;8(6):e65201. doi: 10.1371/journal.pone.0065201 (PMC3675204; doi:10.1371/journal.pone.0065201)
Supplement: Text S1 — Detailed description of methods. (DOC) [file pone.0065201.s005.doc]

**Text S1.**

Detailed Description of Methods

*Animals*. All studies were approved by the Institutional Animal Care and Use Committee at the University of Iowa (PHS Animal Welfare Assurance #A3021-01). We studied littermate LA male and female mice. Beginning at age 2 months, LA mice received an atherogenic “Western” diet (Harlan Teklad #TD88137, 42% of calories from fat, 0.25% cholesterol).

*Experimental strategy*. (See manuscript Figure 1.) Mice were studied at two ages. “Young” Veh-LA mice received injections of vehicle (PBS, 0.1 ml s.c., 3 times per week), and OPG-LA mice received human Fc-OPG ( 50 mg/kg in PBS, s.c., 3 times per week; Amgen Inc., Thousand Oaks, CA), beginning at 2 months of age. At 8 months of age, Young mice underwent echocardiography, followed by euthanasia, blood collection, and histologic studies.

“Older” mice were started on the Western diet at about 2 months of age. At age 6 months, echocardiography was performed to assess pre-treatment aortic valve function. Mice which had already demonstrated decreased aortic valve function, defined as systolic cusp separation ≤ 0.8mm (a total of 4 mice), were excluded from subsequent study. Then, at 6 months of age, 13 Veh mice received injections of vehicle, and 12 OPG mice received OPG at the same dose and frequency as Young mice. At age 12 months, Older mice from each group underwent echocardiography, followed by euthanasia, blood collection, and histologic studies.

*Echocardiography*. Aortic valve function was quantified using a method that we have validated hemodynamically, as described previously.[S1] Mice were lightly sedated with midazolam (0.15 mg s.c.), during which they remained conscious but docile. A 15-MHz (Philips Medical Systems, Bothell, Wash) or 30 MHz (VisualSonics, Toronto) linear-array probe was applied horizontally to the chest. M-mode images of the aorta and aortic valve were obtained at ~1000 /second, with two-dimensional images used for guidance. Doppler interrogation of blood velocity through the aortic valve was not performed because the linear-array probes do not have continuous-wave Doppler capability. All images were acquired and analyzed by operators blinded to genotype and treatment/diet. The systolic valve orifice was measured off-line by an investigator who was blinded with respect to genotype and treatment group, using software designed for that purpose.

*Blood chemistry*. Mice were anesthetized with a lethal dose of inhaled sevoflurane. The heart was exposed via sternotomy, and blood was collected from the inferior vena cava. After centrifugation, the plasma fraction was stored at -80°C for analysis of plasma cholesterol using a colorimetric kit (Wako Diagnostics).

*Histologic studies*. Serial sections, 10 μm thickness, were cut from frozen specimens aligned perpendicular to the plane of the aortic valve. Assessment of the each histologic component was performed in triplicate, utilizing slices from the cusp base, mid-cusp, and distal cusp, which were then mounted on a single slide, imaged, then averaged for each individual histologic component, for each mouse. For each histologic parameter, samples from Veh-LA mice and OPG-LA mice were stained and analyzed in the same setting, so as to avoid artifactual “batch” effects.

Lipid deposition was measured using Oil Red O (Sigma, France). Valve collagen content was assessed using Masson’s Trichrome stain and, in a subset, Picrosirius Red staining. Valve calcification was measured using Alizarin Red stain (Sigma). Immunohistochemical studies of valve tissue were carried out using the following reagents: osterix (Abcam, #22552, 1:75), osteocalcin (1:50; ABBIOTEC, #250483), MCP-1 (1:100; Cell Signaling, #2029).

Methods for measurement of valve superoxide have been described previously.[S1] Briefly, sections 10 μm thick were incubated in 0.002 mmol/L dihydroethidium (DHE) for 30 minutes, followed by fluorescent confocal imaging at 585 nm. Specificity of superoxide was confirmed by incubation of adjacent sections with polyethylene glycol superoxide dismutase (PEG-SOD), and loss of fluorescent signal.

Histologic images were viewed using light microscopy at 4x and 10x magnification (Olympus BX 51 Digital Light Microscope, Olympus, Japan). Tissue content was quantified using Adobe Photoshop CS2 (version 7, Adobe Systems Inc. San Jose, CA) to isolate pixels expressing specific histological staining. Histologic valve cusp area was determined by digital planimetry. For each histologic parameter, data were tabulated as # positive pixels in valve tissue divided by total number of valve pixels. An exception to this procedure was used for quantitation of osterix, which utilized a fluorescent secondary antibody (green, see manuscript Figure 3). For osterix, fluorescence within the borders of valve tissue was quantitated, then normalized to valve cusp area, and expressed in Relative Light Unites (RLU).

*Gene expression*. Total RNA was extracted from the distal thoracic aorta with TrIzol reagent (Invitrogen), and prepared using the RNeasy Mini kit (Qiagen). Reverse transcription reaction (RT) was performed as described previously.[S2] An identical amount of RT product was used for quantitative real-time PCR with a single well of a 96-well plate containing both TaqMan^R^ probes/primers (Applied Biosystems) for target gene (OPG, RANK, RANKL, TRAIL, with FAM fluorophor) and for a house-keeping gene (β-actin, with VIC fluorophor). Expression of target genes was normalized to β-actin. Relative expression levels were obtained using the ΔΔCt method as described previously.[S2]

**Supporting Information References**

S1. Weiss RM, Ohashi M, Miller JD, Young SG, Heistad DD (2006) Calcific aortic valve stenosis in old hypercholesterolemic mice. Circulation 114:2065-2069.

S2. Chu Y, Heistad DD, Knudtson KL, Lamping KG, Faraci FM (2002) Quantification of mRNA for endothelial NO synthase in mouse blood vessels by real-time polymerase chain reaction. Arterioscler Thromb Vasc Biol 22:611-6.
